# Chemical Composition and New Biological Activities of Essential Oil and Hydrosol of *Hypericum perforatum* L. ssp. *veronense* (Schrank) H. Lindb

**DOI:** 10.3390/plants10051014

**Published:** 2021-05-19

**Authors:** Elma Vuko, Valerija Dunkić, Mirko Ruščić, Marija Nazlić, Nela Mandić, Barbara Soldo, Matilda Šprung, Željana Fredotović

**Affiliations:** 1 Department of Biology, Faculty of Science, University of Split, R. Boškovića 33, 21000 Split, Croatia; elma@pmfst.hr (E.V.); dunkic@pmfst.hr (V.D.); mrus@pmfst.hr (M.R.); mnazlic@pmfst.hr (M.N.); nmandic@pmfst.hr (N.M.); 2 Department of Chemistry, Faculty of Science, University of Split, R. Boškovića 33, 21000 Split, Croatia; barbara@pmfst.hr (B.S.); msprung@pmfst.hr (M.Š.)

**Keywords:** *Hypericum perforatum* ssp. *veronense*, essential oil, hydrosol, antiproliferative, antioxidant and antiphytoviral activity

## Abstract

The chemical profile, antiproliferative, antioxidant and antiphytoviral activities of the species *Hypericum perforatum* ssp. *veronense* (Schrank) H. Lindb. (Clusiaceae) were investigated. Free volatiles were isolated and the chemical composition was determined in the lipophilic fraction (essential oil) and for the first time in the water fraction (hydrosol). The aim is to provide phytochemical data for *H. perforatum* ssp. *veronense* useful for distinguishing ssp. *veronense* from ssp. *angustifolium*, as there are taxonomic disagreements between them and the composition of the secretory products may be helpful in this respect. In the essential oil, the most abundant compounds identified were α-pinene and *n*-nonane, while in the hydrosol, myrtenol, carvacrol and α-pinene were the most abundant. Overall, the class of monoterpenes and oxygenated monoterpenes dominated in the EO and hydrosol samples. The essential oil showed high antioxidant activity, in contrast to the antiproliferative activity, where the hydrosol showed exceptional activity against three cancer cell lines: Hela (cervical cancer cell line), HCT116 (human colon cancer cell line) and U2OS (human osteosarcoma cell line). Both the essential oil and hydrosol showed antiphytoviral activity against tobacco mosaic virus infection on the local host plants. This is the first report dealing with biological activities of hydrosol of *H. perforatum* ssp. *veronense*, and the obtained results suggest that this traditional medicinal plant is a valuable source of volatiles with promising antiproliferative, antioxidant and antiphytoviral activities.

## 1. Introduction

*Hypericum perforatum* L. or St. John’s wort (Clusiaceae) is a perennial rhizomatous herb traditionally used in herbal medicine throughout the world. Although various activities of *H. perforatum* extracts have been reported such as antibacterial, antiviral, wound healing, antioxidant, antimicrobial, anti-inflammatory, antinociceptive, antitumor, antiangiogenic, and immunomodulatory activity [1,2,3,4,5,6,7,8,9,10,11], the focus of interest is on its potential as an herbal antidepressant [2]. Flora Croatica Database (FCD) [12] describes two subspecies of *H. perforatum* in Croatia: ssp. *perforatum* L. and ssp. *veronense* (Schrank) H. Lindb. In Italy, Pignatti [13] describes a third subspecies, *angustifolium* (DC.) Gaudin. FCD considers ssp. *angustifolium* to be a synonym of ssp. *veronense* and the Flora of Europe [14] describes only the species *H. perforatum* with two different varieties (var. *angustifolium* and var. *microphyllum*), which points us to a particular taxonomic problem with this species. *H. perforatum* ssp. *veronense* grows in the eumediterranean climate under extreme environmental conditions, such as high solar radiation and drought, in contrast to ssp. *angustifolium*, which grows in more temperate climates. In addition to the described differences in morphological characteristics, such as larger and broader leaves of ssp. *angustifolium* compared to ssp. *veronense* [13], a different phytochemical composition of these subspecies can also be expected due to the different environmental conditions. The term chemophenetics, has recently been proposed for the field of studies aimed at the exploitation of characteristic arrays of specialized natural products of plant taxa [15]. In general, the free volatiles of the genus *Hypericum* have been extensively studied and they also show significant variation in volatile profile. Germacrene D, α-pinene, *β*-caryophyllene, *2*-methyloctane and *n*-nonane were among the major components in essential oils of *H. perforatum* reported by many authors [16]. In this study, the essential oil composition and, for the first time, the hydrosol composition of Croatian *H. perforatum* ssp. *veronense* were investigated, especially with regard to the comparison of volatile components between them. Our results of phytochemical profiling were compared with literature profiles of this species with the aim of providing data useful in distinguishing *H. perforatum* ssp. *veronense* from ssp. *angustifolium*.

In addition to the chemical composition, our team also investigated the biological activities in terms of antiproliferative, antioxidant and antiphytoviral activity of both the essential oil and hydrosol of *H. perforatum* ssp. *veronense*. Due to the difference in solubility in water, the overall composition as well as the biological activity differs between the lipophilic and hydro fractions. Comparison of the efficacy between these fractions may give us an insight into the new biological activities and possible applications of this traditional medicinal plant. To the best of our knowledge, the antiproliferative and antiphytoviral activities of the essential oil of *H. perforatum* have not been studied before, and moreover, this is the first report dealing with these activities of hydrosols of aromatic plant species in general. Hydrosols from various plants are gaining importance in cosmetology, aromatherapy, traditional pharmacy, food industry and plant protection, so their potential use should therefore be further investigated.

The antiproliferative activity of various plant extracts and essential oils has been reported for several *Hypericum* species and their individual constituents, but as mentioned, the same has never been done with hydrosol. The essential oil from *H. hircinum* subsp. *majus* showed moderate antiproliferative activity against human glioblastoma (T98G), human prostatic adenocarcinoma (PC3), human squamous carcinoma (A431) and mouse melanoma (B16-F1) cell lines. The possible mechanism behind the antiproliferative activity in glioblastoma and melanoma cells could be the induction of authophygy [17]. França et al. [18] showed that hexane extract and phloroglucinol derivatives obtained from *H. brasiliense* are potent inhibitors of ovarian (OVCAR-03 and NCIADR/RES) and melanoma (UACC-62) cell division. Three endemic *Hypericum* species from Canary Islands (*H. reflexum*, *H. canariense* and *H. grandifolium*) showed potent cytotoxic activity against human melanoma cell line (A375), breast adenocarcinoma cell line (MDA-MB 231) and colon cancer cell line (HCT116). The authors hypothesize that a synergy between different compounds, rather than the single most dominant compound from the essential oil, is responsible for this activity [19]. The relationship between the potent antioxidant activity of *H. montbretii* and *H. organifolium* leaf and flower extracts and the antiproliferative effect on A549 lung cancer and Hela cervical adenocarcinoma cells was demonstrated by Güzey et al. [20]. Interestingly, all the extracts showed a different mode of antiproliferative action and that too at different concentrations. Probably, the reason for this lies in the differences in the phytochemical profile of the tested extracts.

Plants belonging to the genus *Hypericum* are commonlyused to treat conditions caused by oxidative stress, such as skin wounds, eczema, and inflammation, due to their antioxidant activity [21]. Since literature results showed that phenolic extracts of *H. perforatum* have higher antioxidant activity than many aromatic and medicinal plants such as *Lavandula angustifolia* Mill., *Verbena officinalis* L., *Taraxacum officinale* complex, *Mentha piperita* L. and *Pulmonaria officinalis* L. [22], ORAC and DPPH assays were performed with both hydrosol and essential oil of *H.p.* ssp. *veronense* to gain insight into the antioxidant potential of lipophilic and hydrophilic fractions of this plant species.

Natural antiphytoviral preparations are a new area of research in the field of plant protection against viruses. We hypothesized that the essential oil of *Hypericum* species may have antiphytoviral activity, which is related to findings that essential oils of aromatic plant species, especially sesquiterpene-rich oils, inhibit viral infection on host plants [23,24,25,26,27,28,29]. Moreover, high solar radiation in the eumediterranean climate and the drought are the stressful conditions that *H. perforatum* ssp. *veronense* faces in its natural environment; therefore, the volatiles could help the plant overcome various environmental and biological stressors, including pathogen attack. Although the antiphytoviral activity of hydrosols has not yet been investigated, we assume that, in addition to essential oils, hydrosols can also trigger a response to viral infections in plant systems. This is related to the fact that hydrosols, as condensed water vapors, contain dissolved essential oil components and more polar volatile compounds that are soluble in water [30].

The presented results reveal the chemical composition of the volatile compounds of the lipophilic and water fraction of *H. perforatum* ssp. *veronense* in relation to new biological activities of this traditional medicinal plant. In view of all this, and in addition to the traditional use of *H. perforatum* as a natural antidepressant, the essential oil and hydrosol of *H. perforatum* ssp. *veronense* could be used in crop protection, pharmaceutical industry and in the production of food supplements.

## 2. Results and Discussion

### 2.1. Gas Chromatography and Mass Spectrometry (GC-MS) Analysis of the Free Volatile Compounds from Essential Oils and Hydrosol

The volatile compounds in the essential oil (EO) and hydrosol of *H. perforatum* ssp. *veronense* were analysed by GC-MS analysis and are listed in Table 1 in the order of their elution from the column. The total oil yield was 0.09%, based on the dry weight of the samples. Forty-five compounds, divided into eight classes, and twenty-five compounds, divided into six classes, were identified in the EO and hydrosol, respectively, accounting for 85.25% and 72.21% of the total oil and hydrosol. In terms of compound classes, monoterpenes (24.59%) and oxygenated monoterpenes (25.7%) dominate the EO and hydrosol samples, respectively. α-Pinene is the dominant compound in EO (16.58%) and is one of the more abundant components (8.69%) in the overall hydrosol composition (Table 1). Maggi and Ferretti found that the flower oil of *H. perforatum* L. ssp. *veronense* (Schrank) Ces. from central Italy contains mainly α-pinene (35.6%), followed by 2-methyloctane (16.9%) [31]. In contrast, no monoterpenes were identified in the oil composition of *H. perforatum* ssp. *angustifolium* from southeastern France, and oxygenated monoterpenes accounted for only 2.6% of the total oil composition [32]. Besides the dominant α-pinene, β-pinene (3.67%) and β-thujone (3.24%) stand out among the monoterpenoids in the oil composition of *H. perforatum* ssp. *veronense* (Table 1). However, these compounds were not identified (β-thujone) in the hydrosol composition or had low content (β-pinene) (Table 1). The proportion of the oxygenated monoterpenes myrtenol (12.33%) and linalool (4.56%) is significantly higher in the hydrosol than in the oil sample, as is the proportion of myrcene (2.55%), limonene (2.63%), α-terpineol (3.57%), β-cyclocitral (2.38%) and camphor (2.17%). Oxygenated monoterpenes, with myrtenol as the most abundant hydrosol constituent, are the predominant class of compounds in the overall hydrosol composition (Table 1). The sesquiterpenes in EO consisted mainly of hydrocarbons (17.27%), while the oxygenated fractions were represented by 12.51%. (E)-Caryophyllene (9.52%) and caryophyllene oxide (7.69%) are the major sesquiterpenes in the composition of EO. These two compounds were also identified in the hydrosol, but the proportion was much lower (2.25% and 0.76%, respectively) (Table 1). Compared to our results, the essential oil of *H. perforatum* ssp. *angustifolium* from South Serbia contained a higher proportion of sesquiterpenes (44.4%), with caryophyllene oxide (15.3%) as the main component, and the content of monoterpenes was low (6.2%) [33] similar to *H. perforatum* ssp. *angustifolium* from France [32]. The non-terpenoid compound n-Nonane is the second most important component in terms of total oil composition, accounting for 13.59%; in hydrosol it accounts for 4.29% of the composition (Table 1). This aliphatic compound has been reported by many authors as one of the main constituents in the essential oils of *H. perforatum*, although in the composition of EO of *H. perforatum* from Serbia the percentage of *n*-nonane was much lower [16]. The monoterpenoids carvacrol and thymol are identified as the major phenolic compounds in hydrosol (9.87 and 3.48%, respectively) (Table 1). With respect to the major components previously identified in the oils of *H. perforatum*, two chemotypes of the oil can be roughly distinguished [16]. The essential oils containing germacrene D, (*E*)-caryophyllene and caryophyllene oxide as major components belong to one chemotype group, while the second group comprises the oils dominated by α-pinene and/or 2-methyloctane [16]. According to the literature results, the components listed in the first chemotype group dominate in the volatile profile of *H. perforatum* from Serbia (germacrene D and (*E*)-caryophyllene) and in the volatile profile of *H. perforatum* ssp. *angustifolium* from South Serbia (caryophyllene oxide), but also in the composition of *H. perforatum* ssp. *angustifolium* from France, noting that the very high content of spathulenol in this oil could be due to germacrene D and bicyclogermacrene, which are known fragile molecules that can be converted into spathulenol [16,32,33]. In contrast to ssp. *perforatum* and ssp. *angustifolium* and in view of our results (Table 1), *H. perforatum* ssp. *veronense* from Croatia belongs to the chemotype group comprising the oils dominated by α-pinene. Moreover, in the oil of ssp. perforatum from Italy, β-caryophyllene (24.4%) and 2,6-dimethylheptane (18.2%) were the main components, while the same authors described α-pinene (35.6%) as the main component in the flower oil of ssp. *veronense* [27]. The above results go along with the idea of chemophenetics, a term recently described by Zidorn [15]. The plant organ used for extraction, the phenological stage of the harvested material, the altitude of the growing area, the genotype and different types of biotic and abiotic stressors have already been discussed as factors influencing the chemical variations in *Hypericum* species [16,33,34,35].

### 2.2. Antiproliferative Activity

**Figure 1:**
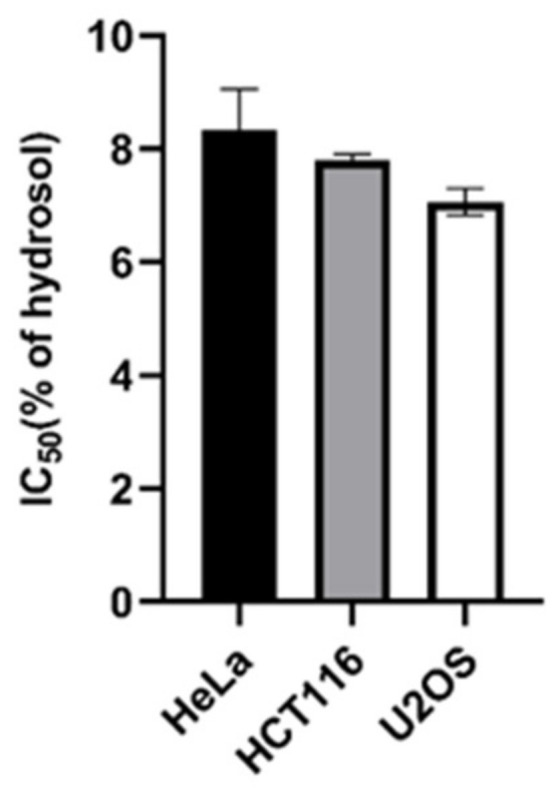
Antiproliferative activity of *H. perforatum* ssp. *veronense* determined by MTS-based cell proliferation assay. Statistical analysis was performed using one-way ANOVA followed by Turkey’s multiple comparison test. Error bars indicate the standard deviation of triplicate analyses.

The influence of essential oil and hydrosol of the species *H. perforatum* ssp. *veronense* on the proliferation of cancer cells has been studied. As far as we know, no one has investigated the antiproliferative effect of the hydrosol of this traditional medicinal plant. In the literature, we found only one study on the cytotoxic activity of the aqueous extract of a species of the genus *Hypericum*, namely *H. scabrum*, against three cancer cell lines: MCF-7, HCT-116 and LNCaP [38]. In contrast to the aqueous extract, the object of our interest was the hydrosol because it contains volatile, water-soluble compounds. Our study showed exceptionally good activity of hydrosol on all three cancer lines tested: Hela (IC_50_ = 8.3%), HCT116 (IC_50_ = 8.81%) and U2OS (IC_50_ = 7.05%) (Figure 1). 

In contrast to hydrosol, the essential oil of *H. perforatum* ssp. *veronense* did not show significant antiproliferative activity. The fact that the essential oil was not tested as a fresh product could cause the evaporation of the main active ingredients and thus the lack of activity. We also cannot exclude that the composition of the oil, which is different from the hydrosol, and the concentrations of its constituents may be the reason for the differences in biological activity, and in this regard further studies of the antiproliferative activity of the fresh essential oil are possible. Among the oxygenated monoterpenes, the hydrosol contains a higher amount of camphor, α-terpineol and β-cyclocitral than the essential oil, and linalool and myrtenol are present as the two most dominant compounds (Table 1). Linalool has shown significant antiproliferative activity on a variety of cancer cells [39,40] including multidrug-resistant human breast adenocarcinoma cells [41]. It appears to play an important role in disrupting improper cell division and maintaining genome stability after genotoxic stress [39]. Myrtenol is a monoterpene compound found in the essential oils of many medicinal plants and has long been used in traditional medicine to treat anxiety, gastrointestinal pain, inflammation and infection [42,43]. There is evidence of its antimicrobial [44] and antioxidant activity [45], but there are no data on its possible antiproliferative activity. In contrast to the essential oil, hydrosol is richer in phenolic compounds such as thymol and carvacrol, which are known to be the most active natural antioxidants. Thymol has been shown to be a compound that can inhibit the division of various cancer cells [46,47,48,49]. Carvacrol is a phenolic monoterpenoid known for its antiangiogenic, analgesic, antioxidant, antimicrobial, and anti-inflammatory properties [50,51,52]. Its antiproliferative activity on liver, lung, colon, and breast cancer cell lines has also been demonstrated [52,53,54,55,56]. Yin et al. [56] suggested that the induction of apoptosis may be the main mechanism of the cytotoxic activity of carvacrol against HepG2 and metastatic breast cancer cells. Several authors also confirmed that thymol induces apoptosis in HL-60 promyeloid cancer cells and human glioblastoma cells [57,58]. As shown below in our study, the antioxidant activity of essential oil is better than that of hydrosol (Table 2). This may be explained by the concentration differences of the phenolic monoterpenoids carvacrol and thymol in the oil and hydrosol (Table 1), which show strong antioxidant power at lower concentrations, changing to prooxidant at higher concentrations [59]. However, this could be the reason for the antiproliferative activity of hydrosol, which is rich in these phenolic compounds that could thus cause oxidative stress and consequently inhibit the division of cancer cells.

The antiproliferative effects of *Hypericum* species around the world have been previously studied in various cancer cell lines using different plant extracts ranging from methanol, ethanol, ethyl acetate, dichloromethane or hexanol to single dominant compounds or mixtures of several compounds. Zorzetto et al. [19] showed that polar extracts and essential oils of *H. reflexum, H. canariense* and *H. grandifolium* strongly inhibited the proliferation of human melanoma cell line A375, breast adenocarcinoma cell line MDA-MB 231 and colon cancer cell line, HCT116. They also tested some individual essential oil compounds such as α-pinene, β-pinene, (*E*)-caryophyllene and *n*-nonane. The individual compounds showed weaker antiproliferative effect on the tested cancer cells than the essential oil. One possible reason for this effect is that the essential oil contains compounds that act synergistically to stop the growth of cancer cells. Crude methanol extracts of southern Brazilian *Hypericum* species fractionated with solvents of increasing polarity (hexane, chloroform and methanol) showed increasing inhibition of the growth of HT-29 human colon cancer cells and H-460 non-small cell lung cancer as the polarity of the fractions decreased [60]. The hexane fractions were found to be the most effective, probably because they are rich in lipophilic phenols, which have already been shown to be potent inhibitors of in vitro cancer cell proliferation [61,62]. The flower extract of H. perforatum L. showed significant growth inhibition and induction of cell death in K562 erythroleukemic cells. In contrast to the extract, pure hypericin, which is one of the predominant compounds of the extract, showed weaker cytotoxic effect, leading to the conclusion that other compounds, not only hypericin, inhibit cancer cell growth, individually or synergistically [63]. Gönenç et al. [64] showed that ethanol extracts of H. perforatum possess antiproliferative activity against human cervical cancer cell line (HeLa), breast cancer cell line (HCC-1937) and osteosarcoma cell line (U2OS). A possible mechanism of the antiproliferative effect of the extracts could involve apoptosis and autophagy. Quassinti et al. [17] also showed that a model of the antiproliferative activity of the essential oil of *H. hircinum* L. subsp. *majus* (Aiton) N. Robson on glioblastoma and melanoma cells could involve autophagy. The authors emphasized the importance of further research to isolate and identify the essential oil compounds responsible for autophagy in these cancer cell lines. Guzey et al. [20] came to a similar conclusion. They confirmed the good antiproliferative activity of several species of the genus *Hypericum*, as well as the apoptotic effect apparently associated with good antioxidant activity. Our results have shown for the first time that, in addition to essential oils and other *Hypericum* extracts, hydrosol of *H. perforatum* ssp. *veronense* has considerable potential as a possible cytotoxic agent. Further studies on other cell lines are required to assess the feasibility of using hydrosol for pharmacological purposes.

### 2.3. Antioxidant Activity

To determine the antioxidant potential, ORAC and DPPH were performed on two extracts of *H. perforatum* ssp. *veronense*, hydrosol and essential oil. The results presented in Table 2 show higher antioxidant activity of essential oil than hydrosol in both ORAC and DPPH methods. Phenolic monoterpenoids are important antioxidant compounds as described by Llana-Ruiz-Cabello et al. [59] who showed that carvacrol and its mixture with thymol exhibited protection against induced oxidative stress at low concentrations; however, at high concentrations they induced rather than prevented oxidative stress. These results could explain the lower antioxidant activity of the hydrosol of *H. perforatum* ssp. *veronense* compared to the essential oil, considering the amount of thymol and carvacrol in these extracts (Table 1). Aazza et al. [65] reported a similar antioxidant activity (282.6 μmol/L) for Salvia officinalis hydrosol. Quassinti et al. [17] tested the antioxidant activity (DPPH) of essential oil for the species *Hypericum hircinum* L. subsp. *majus* and reported an IC_50_ value of 0.68 mg/mL, which is higher than the result we reported. This could be due to the high content of the compound *cis*-β-guaiene, which was the main compound in the essentail oil of *H. hircinum*. Zardi-Bergaoui et al. [66] studied the essential oil of *Pulicaria vulgaris* subsp. *dentata* and reported that the essential oil from the aerial parts had a significantly higher content of *cis*-β-guaiene than the root parts. Moreover, the aerial parts showed higher antioxidant activity, which, considering the content of *cis*-β-guaiene, could explain the higher antioxidant activity described for the species *H. hircinum* L. subsp. *majus*. Bentayeb et al. [67] evaluated the antioxidant activity for several essential oils of plants commonly used as spices. The results (Table 2) showed that the essential oil of *H. perforatum* ssp. *veronense* had higher antioxidant activity than dill (seed), rosemary, basil and lemongrass and comparable activity to thyme essential oil. Previously, Kratchanova et al. [68] reported antioxidant activity for acetone and water extracts of St. John’s wort. Acetone extracts showed higher antioxidant activity (1141 ± 93 μmol/g DW) than water extracts (629 ± 41 μmol/g DW). Napoli et al. [21] reported ORAC values for antioxidant activity of ethanolic extracts of *H. perforatum* of 890 μmol/g DW, which were lower than the acetone extracts reported by Kratchanova et al. It is possible that acetone extracts contain not only phenolic compounds but also other specialized metabolites such as essential oil components that contribute to the higher antioxidant activity. In conclusion, the essential oil of *H. perforatum* ssp. *veronense* exhibits considerable antioxidant activity that deserves further research.

### 2.4. Antiphytoviral Activity

*Hypericum* species from around the world are a source of bioactive essential oils. Since ancient times, essential oils have been used in folk medicine not only as fragrances or preservatives for food, but also for very important biological activities (e.g., antibacterial, antifungal, antiviral, anti-inflammatory) [69]. In addition, there is a wealth of information on the role of essential oils in plant-plant, plant-animal, or plant-insect interactions [70]. We have previously reported that essential oils from different plant species can trigger a response to viral infections in plants [23,24,25,26,27,28,29], but the same has never been shown for hydrosols. We hypothesise that hydrosols, as water solutions of bioactive components during the distillation process of essential oil, are a readily available and safe natural source of bioactive components that can serve as a mixture of active ingredients that can protect plants against viral infections. Thus, in addition to the volatile profile and antiphytoviral activity of the essential oil, the aim of this research was to study for the first time the antiphytoviral activity of the hydrosol of *H. perforatum* ssp. *veronense* and to provide some new data that can lead us towards natural plant protection and the reduction of the use of synthetic agents in the fields. The activity of both the essential oil and hydrosol of *H. perforatum* ssp. *veronense* on the defence response of local host plants to tobacco mosaic virus (TMV) infection was investigated. Local host plants treated with essential oil prior to virus infection significantly reduced the number of local lesions in the early stages of infection (Table 3). In addition to the reduction in the number of local lesions, a delay in the onset of symptoms was observed in the essential oil treated plants. In the control group, the local lesions appeared on the third day post inoculation, while in the essential oil-treated group, a significant number of inoculated leaves had no symptoms at that time (mean value of local lesions for control and treatment was 6.05 and 0.72, respectively) (Table 3). 

**Figure 2:**
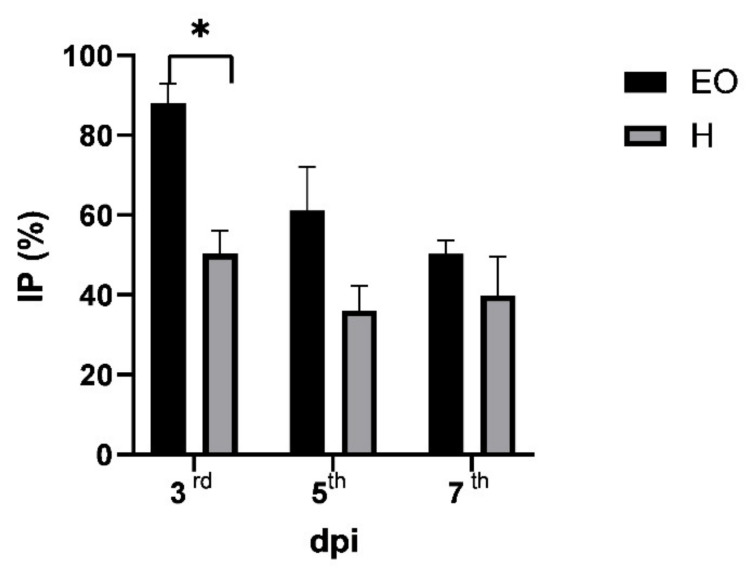
Percentage of inhibition (IP) of local lesions on leaves of tretaed host plants inoculated with tobacco mosaic virus compared with control plants on days 3, 5, and 7 postinoculation (dpi). Prior to inoculation, treated plants were sprayed with essential oil (EO) or hydrosol (H) of *H. perforatum* ssp. *veronense* for two consecutive days. Error bars show the standard deviation of triplicate analyses; significant differences were determined by *t*-test and marked with *.

The inhibition of local symptoms on the third day after inoculation reached a significant value of 88.05% (Figure 2). By the fifth day after inoculation, local lesions also developed on the inoculated leaves of the essential oil treated plants, but the percentage of inhibition compared to the control was still high (61.10%); on the seventh day post inoculation, the percentage of inhibition of local lesions on the leaves of the plants treated with essential oil was 50.33% (Figure 2). Comparing previous results dealing with the antiphytoviral activity of essential oils of different plant species [23,24,25,26,27,28,29] with the current results (Figure 2), we can conclude that pretreatment of local host plants with the essential oil of *H. perforatum* ssp. *veronense* shows a promising activity level, especially in the early stage of infection. Pretreatment with the hydrosol of *H. perforatum* ssp. *veronense* also reduced the development of local lesions on host plant leaves (Table 3), although the efficacy was weaker compared to the oil (Figure 2), possibly related to the differences in the qualitative and quantitative composition between them described in Section 2.1. The delay in the appearance of symptoms was not noticed on the leaves of hydrosol-treated plants compared to the control; local lesions appeared on the third day post inoculation in both groups; nevertheless, the number of local lesions on the leaves of hydrosol-treated plants was significantly reduced compared to the control on the third, fifth and seventh day post inoculation (Table 3), and the percentages of inhibition of local lesions on hydrosol-treated plants were 50.37, 36.10, and 39.87%, respectively (Figure 2). These promising results confirmed our hypothesis about the antiphytoviral activity, not only of the essential oils, but also of the hydrosol of *H. perforatum* ssp. *veronense*. The reported antiphytoviral activity of both essential oil and hydrosol deserves a more detailed analysis in the future and opens new research areas regarding this unexplored bioactivity of essential oils and hydrosols of *Hypericum* species.

## 3. Materials and Methods

### 3.1. Herbal Material

Plants were harvested from the wild in the surroundings of Split, Croatia (43°27′03′′ N, 16°45′13′′ E, 265 m a.s.l.), at full flowering stage from July 2015 to July 2018. The identity of the plant was confirmed by Prof. Mirko Ruščić according to the literature [12,13,14] and voucher specimens of the plant material were deposited at the Faculty of Science, Department of Biology, University of Split, Split, Croatia. For the gas chromatography (GC-FID) and gas chromatography-mass spectrometry (GC and GC-MS) analyses, the samples were air-dried in a single layer in a well-ventilated room for three weeks and protected from direct sunlight. The dried plant material was packed in paper bags and stored in a dry place protected from light until analysis.

### 3.2. GC and GC-MS Analyses

Dried aerial parts (80 g) of the plant material were subjected to hydrodistillation for 3 h in a Clevenger-type apparatus. We collected the fractions of lipophilic (essential oil, EO) and hydrophilic volatile compounds (extracted in the pentane and water fractions, respectively, in the inner tube of the Clevenger apparatus) and stored them in the refrigerator until analysis. Both phases were analyzed by GC and GC-MS. Gas chromatography (GC) was performed using a gas chromatograph (model 3900; Varian Inc., Lake Forest, CA, USA) equipped with a flame ionization detector (FID), a mass spectrometer (model 2100T; Varian Inc.), a nonpolar capillary column VF-5ms (30 m × 0.25 mm i.d., coating thickness 0.25 μm, Varian, Lake Forest, CA, USA) and a polar CP Wax 52 CB (30 m × 0.25 mm i.d., coating thickness 0.25 μm) was equipped. The chromatographic conditions for both fractions were: FID detector temperature 300 °C, injector temperature 250 °C, the carrier gas was helium at 1 mL min^−1^. The conditions for the columns were: VF-5ms (temperature 60 °C isothermal for 3 min, then increased to 246 °C at a rate of 3 °C min^−1^, and held isothermal for 25 min) and for the CP Wax 52 column (temperature 70 °C isothermal for 5 min, then increased to 240 °C at a rate of 3 °C min^−1^, and held isothermal for 25 min). The injected volume was 2 μL and the split ratio was 1:20. The MS conditions were: ion source temperature 200 °C, ionization voltage 70 eV, mass scan range 40–350 mass units [71]. For the hydrophilic fraction, the injection was performed with a headspace injection needle and there was no split ratio (splitless mode). The 2 g of hydrosol was added to the glass bottle and sealed with a metal cap with septum. The headspace needle was injected into the glass bottle sealed with a metal cap with septum. The glass bottle was first placed in 40 °C water with the hydrosol sample and left there for 20 min without the needle to allow the volatile compounds to evaporate from the water. The needle was then injected and left there for 20 min to allow the volatile compounds to adsorb onto the resin needle. The injection needle was then inserted into a GC inlet and left there for 20 min to ensure that all volatile compounds were reabsorbed from the resin into the injection liner. Individual peaks for all samples were identified by comparing their retention indices of n-alkanes with those of authentic samples and literature [36]. The results for all samples were measured in three independent analyzes and expressed as a percentage (%) of each compound (Table 1).

### 3.3. Antiproliferative Analysis

The antiproliferative activity of the essential oil and hydrosol of *H. perforatum* ssp. *veronense* was determined on cancer cells cervical cancer cell line (HeLa), human colon cancer cell line (HCT116) and human osteosarcoma cell line (U2OS) as described by Fredotović et al. [72]. The cells were donated to us by prof. Janoš Terzić from the School of Medicine, University of Split. Antiproliferative activity was determined using the MTS-based CellTiter 96^®^ Aqueous Assay (Promega, Madison, WI, USA). Cells were grown in a CO_2_ incubator at 37 °C and 5% CO_2_ until they reached 80% confluency. Cells were then counted using the automatic handheld cell counter (Merck, Darmstadt, Germany) to check cell number and viability. Cells were seeded in 96-well plates and treated with serially diluted oil and hydrolate. 5000 cells were seeded in each well. Cells were grown for 48 h, after which 20 µL of MTS tetrazolium reagent (Promega) was added to each well. After three hours of incubation at 37 °C and 5% CO_2_, absorbance was measured using a 96-well plate reader (EL808, Bio-Tek, Winooski, VT, USA). Measurements were performed in four replicates for each concentration and IC_50_ values were calculated from three independent experiments using GraFit 6 data analysis software (Erithacus, East Grinstead, UK).

### 3.4. Antioxidant Activity

#### 3.4.1. Oxygen Radical Absorbance Capacity Assay (ORAC)

The assay was performed on a LS55 spectrofluorimeter (Perkin-Elmer, Leatherhead, UK) using 96-well white polystyrene microtiter plates (Porvair Sciences, Leatherhead, UK) according to a method described by Fredotovic et al. [73] and Nazlić et al. [74]. Each reaction contained 180 µL of fluorescein (1 µM), 70 µL 2,2′-Azobis(2-methyl-propionamidine) dihydrochloride (AAPH, Acros Organics, Fair Lawn, NJ, USA) (300 mM), and 30 µL of plant extract or reference standard Trolox (6.25–50 µM) (Sigma–Aldrich, St. Louis, MO, USA). All experimental solutions were prepared in a phosphate buffer (0.075 mM, pH 7.0). The extract of essential oil was prepared in acetone (20 mg/mL). This solution was further diluted 400× with the phosphate buffer for the experiments. For the hydrosol analyses we used total hydrosol diluted 10x. Measurements were performed in triplicate. ORAC values for essential oil were expressed as µmol of Trolox equivalents (TE) per g of essential oil and for hydrosol as µmol of Trolox equivalents (TE) per L of hydrosol.

#### 3.4.2. Measurement of the DPPH Radical Scavenging Activity

The antioxidant capacity of the extracts was assessed by the DPPH method previously outlined by Brand-Williams et al. [75]. This method was adapted due to small amount of the sample to 96-well microtiter plates method previously used by Mensor et al. [76] and Payet et al. [77]. This method uses 96-well microtiter plates for the reaction of reduction of alcoholic DPPH (2,2-diphenyl-1-picrylhydrazyl) solution (Sigma–Aldrich) in the presence of a hydrogen-donating antioxidant. Plant extracts as described in the ORAC method were used (acetone dissolved essential oils and absolute hydrosols). After the final step of adding 100 µL of a methanolic solution of DPPH (200 µM) to each well, the reaction begins and the initial absorbance was measured immediately at 517 nm using MetOH as a blank value. After 30 and 60 min of incubation, the absorbance was measured again and the percentage of DPPH inhibition was calculated according to the following formula by Yen and Duh [78]: % inhibition = ((AC(0) − AA(t))/AC(0)) × 100, where AC(0) is the absorbance of the control at *t* = 0 min, and AA(t) is the absorbance of the antioxidant at *t* = 1 h. All measurements were performed in triplicate. Results were expressed as percentage (%) of inhibition and IC50 values in mg/mL.

### 3.5. Antiphytoviral Activity

#### 3.5.1. Virus and Plant Hosts

Tobacco mosaic virus (TMV) was propagated in a systemic host, *Nicotiana tabacum* L. cv. Samsun. Systemically infected leaves were ground in 0.06 molL^−1^ phosphate buffer, pH 7.0 (1:1, *w/v*), and centrifuged at low speed to prepare the virus inoculum. This inoculum was diluted with inoculation buffer to yield 10–50 lesions per inoculated leaf of the local host plant *Datura stramonium* L. Prior to virus inoculation leaves were dusted with silicon carbide (Sigma-Aldrich). Care was taken to ensure that the experimental plants were as uniform in size as possible.

#### 3.5.2. Antiphytoviral Activity Assay

Essential oil (EO) (final concentration adjusted to 500 ppm) or hydrosol (undiluted) were applied as a spray solution to the leaves of local host plants for two consecutive days prior to virus inoculation and then rubbed with virus inocula (10 plants, 2 leaves per plant) as described by Vuko et al. [23]. Control plants were sprayed with distilled water and inoculated with virus inocula. Lesions were counted on the third, fifth, and seventh day post inoculation and inhibition of local lesions was calculated by comparing the average number of viral lesions on the leaves of treated and control plants according to the formula: IP = [(C − T)/C] × 100, where IP = inhibition of local lesions in %, C = mean number of local lesions on the leaves of the control group; T = mean number of local lesions on the leaves of the group treated with EO/hydrosol. Results are presented as the average of three replicate experiments.

### 3.6. Statistical Analysis

Statistical analysis was performed in GraphPad Prism Version 9 (GraphPad Software, San Diego, CA, USA). All data are expressed as mean ± SD (n ≥ 3). The statistical significance for free volatile compounds, antiproliferative, antioxidant and antiphytoviral activity was assessed by multiple t-test (free volatile compounds, antioxidant activity and antiphytoviral activity), one-way ANOVA followed by Turkey´s multiple comparison test (antiproliferative activity). Differences were considered significant at * *p* < 0.05. Statistical tests were performed separately for lipophilic (essential oils) and hydrophilic fractions (hydrosols).

## 4. Conclusions

Monoterpenes and oxygenated monoterpenes dominate both essential oil and hydrosol samples of *Hypericum perforatum* ssp. *veronense*. Phytochemical profiling of volatile compounds of *Hypericum* species may be useful to distinguish *H. perforatum* ssp. *veronense* from ssp. *angustifolium*. In addition to the pronounced antioxidant activity of the essential oil of *H. perforatum* ssp. *veronense*, the exceptional antiproliferative activity of hydrosol was demonstrated for the first time. Another new finding is that, in addition to essential oils, hydrosols from aromatic plant species have the potential for the development of natural antiphytoviral preparations. The results presented in this study imply further research regarding potential applications of *Hypericum* species.

## Figures and Tables

**Table 1:** Phytochemical composition (% ± SD) of the essential oil (EO) and hydrosol (H) from aerial parts of *Hypericum perforatum* ssp. *veronense*.

Component	RIs	RI ^a^	RIs	RI ^b^	EO (Yield in %)	H (Yield in %)
*Monoterpene hydrocarbons*					24.59	15.38
*α*-Pinene *	932	938	1027	1025	16.58 ± 0.01 ^a^	8.69 ± 0.01 ^b^
Camphene *	946	962	1053	1048	0.59 ± 0.01 ^a^	0.29 ± 0.05 ^b^
Sabinene	969	971	1110	1095	1.22 ± 0.01	-
*β*-Pinene	974	982	1113	1109	3.67 ± 0.01 ^a^	0.34 ± 0.01 ^b^
Myrcene	988	992	1167	1167	0.26 ± 0.02 ^b^	2.55 ± 0.01 ^a^
α-Terpinene	1014	1016	1179	1182	0.79 ± 0.01	-
*p*-Cymene	1020	1021	1265	1268	0.17 ± 0.01	-
Limonene	1024	1032	1196	1204	0.65 ± 0.01	2.63 ± 0.01 ^a^
*(Z)-β*-Ocimene *	1032	1052	1224	1218	-	0.88 ± 0.02
*γ*-Terpinene	1054	1057	1238	1255	0.66 ± 0.01	-
*Oxygenated monoterpenes*					10.84	25.7
Linalool *	1095	1099	1547	1548	0.45 ± 0.01 ^b^	4.56 ± 0.01 ^a^
*β*-Thujone	1112	1121	1435	1438	3.24 ± 0.01	-
Camphor	1141	1151	1515	1499	0.97 ± 0.01 ^b^	2.17 ± 0.01 ^a^
Pinocarvone	1160	1160	1543	1540	0.68 ± 0.01	-
Borneol *	1165	1176	1700	1719	0.55 ± 0.01	-
Terpinen-4-ol	1174	1184	1610	1611	1.26 ± 0.01	-
*α*-Terpineol	1186	1186	1661	1646	0.37 ± 0.02 ^b^	3.57 ± 0.01 ^a^
Myrtenol	1194	1197	1776	1782	0.89 ± 0.01 ^b^	12.33 ± 0.0 ^a^
Verbenone	1204	1204	1720	1705	-	0.69 ± 0.02
*β*-Cyclocitral	1217	1223	1610	1629	0.15 ± 0.01 ^b^	2.38 ± 0.01 ^a^
Linalyl acetate	1254	1252	1553	1553	0.61 ± 0.03	-
Bornyl acetate	1287	1285	1572	1570	0.94 ± 0.01	-
Piperitone oxide	1366	1366	-	-	0.75 ± 0.01	-
*Sesquiterpene* *hydrocarbons*					17.27	5.95
*α*-Copaene	1374	1377	1482	1484	0.23 ± 0.01 ^b^	0.84 ± 0.01 ^a^
*β*-Bourbonene	1387	1383	1500	1508	0.73 ± 0.05	-
(*E*)-Caryophyllene *	1417	1424	1598	1585	9.52 ± 0.01 ^a^	2.25 ± 0.01 ^b^
allo-Aromadendrene	1458	1465	1660	1662	-	1.56 ± 0.01
Germacrene D	1484	1481	1708	1692	1.83 ± 0.01	-
Viridiflorene	1496	1496	1698	1697	1.67 ± 0.01 ^a^	0.37 ± 0.03 ^b^
Bicyclogermacrene	1500	1500	1734	1718	1.47 ± 0.01 ^a^	0.54 ± 0.01 ^b^
*β*-Bisabolene	1505	1494	1728	1729	0.85 ± 0.01 ^a^	0.39 ± 0.01 ^b^
*δ*-Cadinene	1522	1517	1757	1745	0.97 ± 0.01	-
*Oxygenated sesquiterpenes*					12.51	7.54
Spathulenol	1577	1577	2106	2101	2.28 ± 0.01 ^b^	4.93 ± 0.01 ^a^
Caryophyllene oxide *	1582	1581	1954	1955	7.69 ± 0.01 ^a^	0.76 ± 0.01 ^b^
*γ*-Eudesmol	1630	1632	2166	2135	0.87 ± 0.01 ^a^	0.47 ± 0.02 ^b^
*α*-Cadinol	1652	1655	2210	2208	0.67 ± 0.05	-
*α*-Bisabolol	1685	1688	2168	2116	0.76 ± 0.02	-
*α*-Bisabolol oxide	1749	1748	-	2511	0.24 ± 0.02 ^b^	1.38 ± 0.01 ^a^
*Phenolic compounds*					1.69	13.35
Thymol *	1289	1290	2198	2198	0.14 ± 0.01 ^b^	3.48 ± 0.01 ^a^
Carvacrol *	1298	1298	2239	2239	1.37 ± 0.01 ^b^	9.87 ± 0.01 ^a^
Eugenol *	1356	1370	2173	2175	0.18 ± 0.03	-
*Aliphatic compounds*					13.86	4.29
*n*-Nonane	900	900	-	1011	13.59 ± 0.01 ^a^	4.29 ± 0.01 ^b^
*1*-Octen-*3-ol*	974	974	1442	1452	0.27 ± 0.01	-
*Diterpenes*					0.26	-
Phytol	1942	1964	2622	2622	0.26 ± 0.03	-
*Hydrocarbons*					4.23	-
Hexadecanoic acid	1959	1959	-	2476	1.28 ± 0.01	-
Docosane *	2200	2200	2200	2200	0.57 ± 0.02	-
Hexacosane *	2600	2600	2600	2600	1.59 ± 0.01	-
Heptacosane *	2700	2700	2700	2700	0.79 ± 0.01	-
*Total identified (%)*					85.25	72.21

Retention indices (RIs) were determined relative to a series of *n*-alkanes (C_8_–C_40_) on capillary columns VF5-ms (RI ^a^) and CP Wax 52 (RI ^b^); RI, identification by comparison of RIs with those listed in a homemade library, reported in the literature and/or authentic samples; comparison of mass spectra with those in NIST02 and Wiley 9 mass spectral libraries [36,37]; * co-injection with reference compounds; - not identified; SD standard deviation of triplicate analysis; significant differences were determined using multiple *t*-test. ^a^, ^b^ Mean values with different superscripts indicate a statistically significant difference between the data from EO and the H sample (*p* < 0.05).

**Table 2:** Antioxidant potential of *Hypericum perforatum* ssp. *veronense* essential oil and hydrosol determined by ORAC and DPPH method.

	*H. perforatum* ssp. *veronense*	
Antioxidant Assay	Essential Oil	Hydrosol
ORAC (Trolox eq)	2347.65 ± 119.28 ^a^	240.34 ± 7.59 ^b^
DPPH (% inhibition)	44.03 ± 0.74 ^a^	11.88 ± 1.4 ^b^
DPPH (IC_50_)	23.07 ± 0.49	-

ORAC, oxygen radical absorbance capacity, results for EOs expressed as μmol of Trolox equivalents (TE) per g of EO (10 mg/mL) and for hydrosols as μmol of Trolox equivalents (TE) per L of the total (undiluted) tested hydrosol sample; DPPH, IC_50_ expressed in mg/mL for EOs; “-“ could not be calculated. ^a^, ^b^ Mean values with different superscripts indicate statistically significant difference between control and essential oil/hydrosol treatment data (*p* < 0.05).

**Table 3:** Number of local lesions (LLN) on leaves of treated host plants inoculated with tobacco mosaic virus and on leaves of control plants (C) on days 3, 5, and 7 postinoculation (dpi). Prior to inoculation, treated plants were sprayed with essential oil (EO) or hydrosol (H) of *H. perforatum* ssp. *veronense* for two consecutive days. SD, standard deviation of triplicate analysis; significant differences were determined by *t*-test. ^a^, ^b^ Mean values with different superscripts indicate statistically significant difference between control and essential oil/hydrosol treatment data (*p <* 0.05).

dpi		LLN ± SD		LLN ± SD
3rd	C	6.05 ± 2.19 ^a^	C	9.65 ± 3.77 ^a^
	EO	0.72 ± 0.33 ^b^	H	4.93 ± 2.29 ^b^
5th	C	16.64 ± 3.25 ^a^	C	19.00 ± 3.24 ^a^
	EO	6.24 ± 0.64 ^b^	H	12.05 ± 1.60 ^b^
7th	C	19.38 ± 6.05 ^a^	C	28.55 ± 3.82 ^a^
	EO	9.50 ± 2.32 ^b^	H	17.34 ± 4.50 ^b^

## Data Availability

All data is contained within the article.
